# Potential Application of Combined Therapy with Lectins as a Therapeutic Strategy for the Treatment of Bacterial Infections

**DOI:** 10.3390/antibiotics10050520

**Published:** 2021-05-02

**Authors:** João Victor de Oliveira Santos, Ana Lúcia Figueiredo Porto, Isabella Macário Ferro Cavalcanti

**Affiliations:** 1 Laboratory of Immunopathology Keizo Asami (LIKA), Federal University of Pernambuco (UFPE), Recife 50670-901, Pernambuco, Brazil; joao.oliveirasantos@ufpe.br; 2 Department of Morphology and Animal Physiology Animal, Federal Rural University of Pernambuco (UFRPE), Recife 52171-900, Pernambuco, Brazil; analuporto@yahoo.com.br; 3 Academic Center of Vitória (CAV), Laboratory of Microbiology and Immunology, Federal University of Pernambuco (UFPE), Vitória de Santo Antão 55608-680, Pernambuco, Brazil

**Keywords:** proteins, synergism, bacteria, biofilm

## Abstract

Antibiotic monotherapy may become obsolete mainly due to the continuous emergence of resistance to available antimicrobials, which represents a major uncertainty to human health. Taking into account that natural products have been an inexhaustible source of new compounds with clinical application, lectins are certainly one of the most versatile groups of proteins used in biological processes, emerging as a promising alternative for therapy. The ability of lectins to recognize carbohydrates present on the cell surface allowed for the discovery of a wide range of activities. Currently the number of antimicrobials in research and development does not match the rate at which resistance mechanisms emerge to an effective antibiotic monotherapy. A promising therapeutic alternative is the combined therapy of antibiotics with lectins to enhance its spectrum of action, minimize adverse effects, and reduce resistance to treatments. Thus, this review provides an update on the experimental application of antibiotic therapies based on the synergic combination with lectins to treat infections specifically caused by multidrug-resistant and biofilm-producing *Staphylococcus aureus*, *Escherichia coli*, and *Pseudomonas aeruginosa*. We also briefly discuss current strategies involving the modulation of the gut microbiota, its implications for antimicrobial resistance, and highlight the potential of lectins to modulate the host immune response against oxidative stress.

## 1. Introduction

Worldwide, 57 million deaths are reported per year, of which 14.9 million are attributed to infectious diseases [1,2]. For years, clinical therapeutic success was achieved; however, the culture of indiscriminate administration of antibiotics aimed at human health and in agriculture has enabled the dissemination and the acceleration of microbial resistance, which has allowed them to evade the actions of the immune system and drugs, becoming a challenge for current drug therapy [3]. The spread of antimicrobial resistance (AR) represents a major challenge and threat to the future of therapies for various infections [4,5].

In recent years, there has been increasing knowledge of the impact that antibiotics can have on the intestinal microbiota and host immunity response, and how the balance of gut microbiota is essential to regulate numerous aspects of human physiology [6]. The imbalance of the microbiota is related to the loss of the protective effects of the microbiota against pathogens; several metabolic disorders, such as oxidative stress; and the expansion of the host resistance gene pool, known as “resistome”, which can act as an amplifier of antimicrobial resistance [7].

In addition to resistance, bacteria have virulence factors that allow infection to persist, such as biofilms [8]. Biofilm can be defined as complex communities, formed by different microbial cell types, immersed in an extracellular polymeric matrix composed of exopolysaccharides (EPS), proteins, lipids, and DNA adherents to a biotic or abiotic surface [9]. Biofilm-producing microorganisms are responsible for causing most human bacterial infections, as biofilm promotes a protective barrier between bacteria and the environment, making bacteria highly resistant to antimicrobials, contributing significantly to the failure of antimicrobial therapy [10].

In this period of resistance, the medical–scientific community has its efforts focused on the discovery of new drugs or alternative methods of treatment. Unfortunately, it is not possible to guarantee that the new drugs will be effective in the long term to cure such diseases and that the eventual development of resistance to them will not happen, and they may become ineffective in a few years’ time [11,12]. Therefore, innovative and effective approaches are needed to better deal with these diseases. Among the strategies that have been explored, the use of combined therapy has proven to be an emerging and effective option [3].

Monotherapies represent the first-line therapy regimen since the mid-twentieth century, since the first cases of resistance began to become evident: penicillin by *Staphylococcus aureus* in 1928 [13] and streptomycin by *Mycobacterium tuberculosis* in the mid-1960s [14,15]. In addition, monotherapy has some disadvantages, such as limited spectrum, high cost, and the probability of acquired resistance [16]. The toxicity of certain agents used in the clinic, and the possibility of evolution and acquisition of resistance, has led combined therapy to become a first-line treatment for some infections, with a better efficacy and long-term prognosis compared to monotherapy [3,17].

Combination therapy assesses the simultaneous effect of two or more active compounds, usually referred to as a “drug cocktail”. This therapy aims to obtain an overall effect greater than the addition of its individual effects [18]. Combination therapy has advantages, such as expanding the spectrum of action, less chance of developing resistance, an additive or even synergistic effect, decreased side effects, and the possibility of overcoming drug resistance [3,19].

According to the World Health Organization (WHO), combination therapy has been used regularly as a therapy regimen in infectious diseases such as HIV/AIDS and tuberculosis due to its ability to reach different characteristics of the disease, and because it is the most efficient way to avoid resistance [12,20]. There is growing interest in the use of biomolecules with potential therapy for these comorbidities. This interest promoted the direction of research aimed at the search for alternative therapies based on plants, an inexhaustible source of several compounds with therapeutic properties [21]. Thus, the objective of this article was to review, based on studies published between 2015 and 2021, the potential of therapies combined with lectins against bacterial infections, and complementary strategies to overcome antibiotic resistance and oxidative stress through the modulation of gut microbiota.

## 2. Biological Activities of Lectins

Lectins constitute a diverse class of proteins or glycoproteins of non-immune origin, which have at least one carbohydrate recognition domain (CRD), and which bind in a reversible and specific way to mono-, oligo-, or polysaccharides, without modifying their covalent structure [22].

The ability of lectins to recognize carbohydrates present on the cell surface of microorganisms has sparked interest in the possible applications of this biomolecule. They participate in many biological processes such as cell–cell recognition, host–pathogen interactions, cell growth, cell communication, cell adhesion and migration, apoptosis, immunomodulation, mitogenic induction, cancer metastasis, and differentiation [23,24,25,26,27,28]. Thus, lectins are considered one of the most versatile groups of proteins used in biological processes and biomedical research [29].

The application of this biomolecule has advantages such as preventing the initial stage of adhesion, a fundamental step for establishing the infectious process; less propensity for develop resistance to these molecules, due to the different mechanism of action of lectins compared to the antimicrobials used in the clinic; potentiation of antimicrobial activity; better patient tolerance; and lower costs due to being renewable by nature [30,31,32].

### 2.1. Antibacterial Activity of Lectins

The antibacterial activity of lectins against Gram-positive and Gram-negative bacteria results in the agglutination or inhibition of cell growth through its interaction with components of the bacterial cell wall, such as carbohydrates, teicoic acid, lipopolysaccharides, and peptideoglycans [33]. They can also act by inhibiting cell growth through different mechanisms such as altering cell permeability, reducing nutrient absorption, pore formation and consequent extravasation of extracellular content, and/or through interaction with membrane receptors that promote intracellular responses [34]. The recognition of the specificity of a lectin to certain carbohydrates can help block the interaction of the bacteria with the host cells, thus preventing future infections [35].

Although, in the last few years, there has been a significant increase in the number of publications referring to lectins with antibacterial and antibiofilm potential (Table 1), there are still no drugs derived from them in clinical use. There are already several reports that some plant derivatives can increase the activity of drugs used against pathogenic bacteria [36,37,38]. Through the association between natural products and conventional antibiotics, synergistic interactions can be an effective strategy to overcome bacterial resistance [12]. However, so far, few studies have considered the assessment of possible synergism between lectins and conventional antibiotics (Table 2).

An study conducted by Moura and collaborators [45] evaluated the anti-staphylococcal effect of lectins isolated from the bark (MuBL), heartwood (MuHL), and leaves (MuLL) of *Myracrodruon urundeuva*, as well as the interaction effect of the same ones associated with cefoxitin and cefotaxime. MuBL, MuHL, and MuLL presented bacteriostatic (MIC = 12.5–50 µg/mL) and bactericidal (MBC = 100 µg/mL) effects against *S. aureus* NCTC 8325 and MRSA clinical isolates. A synergistic effect was observed in all combinations, presenting the greatest MIC reduction at 14-fold with the combinations MuBL–cefoxitin, MuLL–cefoxitin, MuBL–cefotaxime, and MuLL–cefotaxime against CA-MRSA. An exception was the MuBL–cefoxitin combination, which showed an additive effect against *S. aureus* NCTC 8325 isolate [45]. The lectin extracted from the leaf of *Schinus terebinthifolia* (SteLL) presented synergistic activity when combined with ciprofloxacin against *S. aureus* NCTC 8325 [50].

Procópio and collaborators [47] evaluated the anti-staphylococcal effects of lectin extracted from the leaves of *Calliandra surinamensis* pinnulae (CasuL) against bovine and caprine mastitis isolates, and the synergistic effect of the CasuL–tetracycline combination was proven against *S. aureus,* and CasuL–ampicillin against *Staphylococcus* sp., with a fourfold reduction in the MIC value of the drug [47]. The synergistic effect of the lectin ApuL extracted from the inflorescence of *Alpinia purpurata* with oxacillin against two MRSA strains and ApuL with ceftazidime against MDR *P. aeruginosa* [48] was also demonstrated. Since its emergence, *S. aureus* represents one of the main global causes of nosocomial infections and the main bacterial species that causes mastitis in dairy animals [47,51]. Its diversity of virulence factors, such as biofilm production, in addition to the different resistance profiles such as MRSA and vancomycin-resistant *S. aureus* (VRSA), made it possible to adapt to different environments, which demonstrates its versatility in different epidemiological contexts [52].

Da Silva and collaborators [46] investigated the antimicrobial activity of lectin extracted from the sarcotesta of *Punica granatum* (PgTeL) against *E. coli* producing β-lactamase (CTX-M, CMY, and MBL) isolates, as well as the possible interaction with different drugs. In this study, several interactions had a synergistic effect against some isolates, among them, PgTeL–ceftazidime against four isolates of ESBL-positive *E. coli* and one isolate of MBL-positive *E. coli*, PgTeL–ampicillin against an ESBL-positive isolate, PgTeL–carbenicillin against an ESBL-positive isolate, and PgTeL–cefuroxime against three ESBL-positive isolates. The combination allowed for a 4- to 128-fold reduction in the MIC values of ceftazidime, 33-fold of ampicillin, 16-fold of carbenicillin, and 256- to 1000-fold of cefuroxime [46]. In another study it was possible to verify that the combination of a lectin obtained from the strain of *Acinetobacter baumannii* with ceftazidime against *S. aureus* and *E. coli* showed a significant increase in antibacterial activity compared to monotherapy, with an eightfold reduction in MIC against both microorganisms [49].

Infections caused by ESBL-positive strains are currently reported on almost every continent [53]. β-lactamases represent one of the main mechanisms of resistance to β-lactams and third generation cephalosporins in Gram-negative bacteria [54]. The other therapeutic option is the carbapenems, one of the choices for the therapy of MDR bacteria, which increases the selective pressure on the appearance and dissemination of strains producing carbapenemases [55]. In view of the limited spectrum of antimicrobials, administration combined with lectins has shown to be a promising option [21].

Santos et al. [56] published two studies recently focused on assessing lectin antimicrobial activity and its association with drugs. In the first study, the lectin extracted from the seeds of *Dioclea violet* (DVL) was evaluated, and although it did not present any clinical activity when evaluated alone (MIC ≥ 1024 μg/mL), DVL–gentamicin combination increased the antibiotic activity expressively, reducing the gentamicin MIC from 50.8 to 10.1 μg/mL against MDR *S. aureus*, and from 32 to 12.7 μg/mL against MDR *E. coli* [56]. In the second study, similar results were observed when Santos et al. [57] evaluated the lectin extracted from *Vatairea macrocarpa* (VML) seeds (MIC ≥ 1024 μg/mL); furthermore, when combined with gentamicin, norfloxacin, and penicillin, an increase in antibiotic activity was also seen, with an MIC reduction of 512 to 128 μg/mL, 40.3 to 32 μg/mL, and 512 to 406.4 μg/mL, respectively, against MDR *S. aureus* [57].

Silva and collaborators [32] obtained similar results with the lectin extracted from the seeds of *Parkia platycephala* (PPL) against MDR *S. aureus*. PPL did not show antibacterial activity (MIC ≥ 1024 μg/mL), but PPL reduced the gentamicin MIC from 64 to 25.4 μg/mL. The association of lectin extracted from the seed of *Canavalia ensiformis* (ConA) with gentamicin promoted a reduction of 80% and 37.5% of the MIC value against MDR *S. aureus* and MDR *E. coli*, respectively, while ConA did not show antibacterial activity (MIC ≥ 1024 μg/mL) when administered alone [44]. Gentamicin represents an antibiotic of the aminoglycoside class, with a broad spectrum of antibacterial action, used to treat nosocomial infections caused by Gram-negative and staphylococcal agents, and can be used synergistically with β-lactam for greater treatment coverage [58,59]. Studies have demonstrated a positive modulation of the antibiotic activity of aminoglycosides based on the combination with natural products, being a promising alternative for decreasing bacterial resistance [58,60].

In all reports found, it was observed that lectins intensified the therapeutic effect of commercial antimicrobials, promoting a reduction in MIC. Based on these findings, the lectin could act as an adjuvant when combined with antibiotics in the treatment of bacterial infections.

### 2.2. Antibiofilm Activity of Lectins

The production and cellular release of mature biofilms in medical devices is a constant concern in hospital environments due to the possibility of them becoming a new source of local and systemic infections [61]. In addition, the recurrence of the infectious process often occurs due to the presence of biofilms or even originates from primary infections associated with biofilms [62].

The spread of MDR strains to almost all classes of antimicrobials available worldwide, associated with a decrease in the production/introduction of new effective antimicrobial agents, both for the prevention and treatment of infections caused by biofilm-producing microorganisms, represents a major global health challenge, especially in underdeveloped countries, where infectious diseases are responsible for around 50% of deaths [63,64].

The adhesion and colonization of the surfaces constitute preliminary steps in the development of the infection, but the recognition of the carbohydrates present on the bacterial cell surface could prevent the adhesion to the host cells and to the surfaces of medical devices, making the formation of bacterial biofilm infeasible, in addition to being able to eradicate the preformed biofilms [34,65].

Although the antibiofilm potential of lectins has already been evidenced previously (Table 3), until now, only two articles have verified the interaction of lectins with drugs, meeting the objective of the present study (Table 4).

Fu et al. [69] evaluated the antibiofilm potential of a plasma lectin from *Tachypleus tridentatus* hemolymph (rHPLOE). This lectin was able to inhibit the biofilm formation of *P. aeruginosa* PA14, as well as to disperse the preformed biofilm. The combined therapy with azithromycin and cephalexin was also tested against the preformed biofilm of *P. aeruginosa*, and both combinations were able to inhibit the formation of the biofilm in a concentration-dependent association. From the total protein assay, it was observed that both combinations promoted a significant reduction in the levels of biofilm proteins. This change can destabilize the structure of the mature biofilm, facilitating the action of an antimicrobial. The eradication of biofilms with commercial antimicrobials is a complicated task, and one of the reasons is that the concentration required to eradicate the biofilm can reach a thousand times more than is necessary to eliminate planktonic bacteria of the species [37,70,71,72].

The second study conducted by Procópio and collaborators [47] evaluated the effects of CasuL lectin and the combinations of CasuL–tetracycline and CasuL–ampicillin against *S. aureus* isolated from bovine and caprine mastitis. CasuL showed antibiofilm activity against only one *S. aureus* strain, and all combinations promoted the inhibition of biofilm formation. In front of the Ssp01 isolate, CasuL did not show antibiofilm activity, and against Ssp6PD, CasuL and antibiotics showed no activity; on the contrary, they stimulated the development of biofilm. However, when combined, they reached 60% of inhibition against these isolates. The combination allowed not only a significant result, but also the reduction of the concentration required for each antimicrobial to inhibit this virulence factor. The combination of lectins with conventional antimicrobials can act to increase the spectrum of activity of the drug, reducing or leading to the absence of side effects due to the administration of lower doses, in addition to reducing the possibility of the emergence of resistance and minimizing the impact on therapeutic options in the long term [37].

## 3. Microbiota in Host Health

The human intestinal microbiota consists of a dynamic community of microorganisms, commensals, symbiotics, and pathogens in balance [73]. It is estimated that it is colonized by about 10^14^ bacterial cells, which have essential functions to ensure the health of the host [74]. Despite their diversity, most of the bacteria present in the human digestive tract belong to the phyla Bacteroidetes, Firmicutes, Proteobacteria, and Actinobacteria [75].

Several physiological and immunological processes are influenced and regulated by the microbiota, such as energy homeostasis, metabolism, endocrine signaling, and intestinal epithelial health, among others [76]. However, the microbiota is dynamic and subject to complications in the face of everyday life changes, such as in diet, geography, medical interventions (antibiotics), and comorbidities [73]. These changes can disturb the microbiome, leading to intestinal dysbiosis, either due to the profound or transitory loss of microbial diversity, or the acquisition of pathogenic characteristics by symbiotic microorganisms, resulting in a greater susceptibility to pathogenic invasion and systemic spread by commensal microorganisms [76,77].

In the hospital context, one of its most important functions is the protection against enteric bacterial pathogens, preventing nosocomial infections, since the intestine represents a large reservoir of opportunistic microorganisms [75]. In recent years, studies have shown several implications of the microbiota in the face of indiscriminate and excessive use of antibiotics, such as the increased rates of antimicrobial resistance, expansion of the host resistance gene pool, and loss of the gastroprotective effect of the microbiota, thus increasing the risk of translocation through of the intestinal barrier to other sites, and greater susceptibility to systemic and recurrent infections [6,7].

In addition, antibiotic therapy can lead to the selection of MDR bacteria from its own microbiota, which represents a clinical challenge given the greater risk of therapeutic failure associated with a prolonged hospitalization, and that patient could become a new source of patient–patient infection transmission, which could be fatal for immunocompromised individuals whose commensal microbiota can no longer guarantee protection against colonization by exogenous bacteria [78].

Therefore, different strategies based on the modulation of the composition of the intestinal microbiota to combat antimicrobial resistance has been explored [79]. This modulation is performed mainly through the administration of prebiotics; probiotics; and, more recently, fecal microbiome transplantation (FMT) [6].

### 3.1. Gut Microbiota Modulation

#### 3.1.1. Prebiotics and Probiotics

Prebiotics are nondigestible food components (polysaccharides, oligosaccharides, fibers), which selectively act on the proliferation and activity of beneficial microorganisms present in the colon [80]. Prebiotics induce the reduction in luminal pH, avoiding the adhesion of pathogens, which activate regulatory T cells (Tregs) that generate an anti-inflammatory response and short-chain fatty acids (SCFAs) as a final product, which act as nutrients for the enteric epithelium [76,81].

Probiotics are live microorganisms that, when administered in an adequate amount, confer benefits to the health of the host. In addition to presenting antimicrobial activity against pathogens, it can also cause a reduction in intestinal permeability; increased secretion of mucin and IgA, both involved in the protection of mucous membranes; stimulation of defensins to prevent colonization of pathogens; and increased tolerance of the immune system against commensal pathogens. *Lactobacillus* and *Bifidobacterium* are the anaerobic bacteria most used in probiotics beneficial to human health [6,82]. The efficacy of probiotics in eradicating the intestinal transport of vancomycin-resistant Enterococci (VRE) [82] and colonization reduction of the gastrointestinal tract by MRSA has already been demonstrated when *Lactobacillus rhamnosus* was administered [83]. It is also hypothesized that probiotic species may lead to the reduction of MDR organisms and antibiotic resistance genes (ARGs) present in the intestinal microbiota [84]. Prebiotics and probiotics can also be administered in combination, known as a symbiotic product, due to the synergistic effect promoted by the combination [80].

#### 3.1.2. Fecal Microbiome Transplantation (FMT)

In view of the AR scenario, FMT has stood out as one of the most effective alternatives for infection control, flora restoration, and for the selective eradication of MDR bacteria present in the intestinal microbiota [7]. It comprises the transfer of processed feces from a healthy donor to the colon of a diseased recipient. This transfer can be performed through oral capsules, parenterally, endoscopically, and/or through colonoscopy administration [75,79].

The determinant of its benefits is not yet known, it is believed that it is due to the addition of viable diners capable of restoring intestinal dysbiosis, or by the translocation of viruses, proteins, vitamins, SCFAs, or the combination of these, which play a fundamental role in reversing intestinal imbalance [7,79].

FMT is recommended by the Infectious Diseases Society of America (IDSA) and the Society for Healthcare Epidemiology of America (SHEA) as a therapeutic alternative for patients with recurrent *Clostridioides difficile* infection (CDI) when antibiotic therapy fails [7]. Randomized clinical trials, have already achieved cure rates of an average of 80% after FMT in these patients, and the results are significantly more effective when compared to treatment by fidaxomycin and vancomycin [84,85]. Reduction in the frequency of ARGs and/or colonization by MDR bacteria has already been reported in immunocompetent and immunocompromised patients colonized with carbabenemase-positive and ESBL-positive Enterobacterales after TMF [6,86]. However, standardization and careful selection of fecal samples is essential since ARGs can be acquired through donor feces through FMT [87].

Therefore, strategies aimed at modulating the intestinal microbiota should be optimized and investigated, given their potential to reduce and prevent colonization by MDR bacteria, especially in cases where the treatment of choice has not been effective, thus providing a possible alternative to conventional antibiotic therapy in the treatment of infections, a key issue in fight AR.

#### 3.1.3. Strategies to Combat Oxidative Stress Based on Gut Microbiome Modulation

While an overabundance of symbiotic microorganisms resides in our body, it protects us from a range of exogenous pathogens. Therefore, there must be a balance between the activation of the immune system and tolerance to its own components [88]. The genetic and immunological history of the human beings in the face of different exposures, especially overexposure to antimicrobials, is responsible for shaping the microbial pattern and its function [6].

The microbiota is also sensitive to diet, in which chronic consumption of fatty foods and excessive alcohol intake causes the increase of oxidative stress and inflammatory state, leading to intestinal dysbiosis, and an increased risk of cardiovascular disease [89], metabolic syndromes, insulin resistance [90], and even cancer [91].

Oxidative stress corresponds to the imbalance between the production of reactive oxygen species (ROS) and their removal by protective mechanisms. It results from the excessive production of ROS, or the limitation of defense systems [92]. Despite not being the primary cause of certain diseases, its effect extends to deeper layers of the intestinal wall, which can trigger inflammatory events in obesity, diabetes, and hypertension, given its systemic dissemination [93,94]. Prolonged state of oxidative stress leads beyond to the imbalance of the human microbiota, but also to the programmed death of hepatocytes, due to the activation of resident macrophages [95].

The ROS defense system comprises enzymatic and non-enzymatic antioxidants. The non-enzymatic mechanism involves endogenous (metabolites) and exogenous (nutrients) antioxidants [96]. Among endogenous products, lectins stand out for their ability to modulate the innate immune system, mediating reactive oxygen and nitrogen species, and the adaptive immune system, through the production of cytokines [97]. The role of lectins in guaranteeing the symbiotic interaction between symbiotic plants and microorganisms, caused interest in investigating their possible gastrointestinal effect in vivo using murine models [98]. The adherence capacity of phytohemagglutinin (PHA), lectin extracted from red beans *Phaseolus vulgaris*, was observed, allowing for the colonization of *E. coli* and species of *Streptococcus* [99]. The mushroom lectin, *Agaricus bisporus* (ABL), showed an inhibitory and antiproliferative effect on macrophages, in addition to a reduction in vitro of nitric oxide (NO) production by peritoneal macrophages [25]. Galecin-3 acts in immunomodulatory processes by the prevention of cell apoptosis through mitochondrial protection and inhibition of ROS production [100].

Nutracenics are exogenous compounds derived from food (fruits, vegetables, mushrooms), which have a similar effect to prebiotics in the positive modulation of the microbiota, favoring strains of *Lactobacillus* and *Bifidobacterium*, acting in the prevention and treatment of diseases [101,102].

Polyphenols (PPs) have stood out as one of the most important natural antioxidant bioactives. They are secondary metabolites of plants, and studies indicate that PPs modulate the microbiota through prebiotic effects, the neutralization of free radicals, the reduction of cellular apoptosis via the modulation of mitochondrial dysfunction, and the inhibition of pathogenic intestinal bacteria [96,103]. The microorganisms present in the microbiota act in the metabolization of high molecular weight PPs into more active phenolic metabolites, which significantly influence the structure and function of the community [104]. Another known source of nutraceuticals is grapes, due to their high content of flavonoids, which have antioxidant and anti-inflammatory effects, providing neuro- and cardioprotection, as well as resveratrol, also found in grapes [105].

In conclusion, the combination of traditional drugs and compounds of natural origin, such as lectins, optimizes the inflammatory response to the increasing pressure of oxidative stress, being a possible alternative to prolonged administration of medications such as anti-inflammatories and antibiotics.

## 4. Conclusions

The traditional antibiotic monotherapies do not seem to be a sustainable long-term solution for the treatment of infections caused by resistant bacteria, as they are active in limited spectra and can be ineffective within several years. Antibiotics can also promote an imbalance in the diversity and function of the human microbiome, wich can lead to intestinal dysbiosis. Consequently, the use of a combination therapy of antibiotics with natural products, such as lectins, has become an emerging area of interest in the scientific community as a way to overcome multidrug resistance, and a promising strategy in the modulation of the microbiota to combat oxidative stress. Its stands out for having greater bioavailability, which facilitates the screening process to ensure its non-toxicity through in vivo studies and reduces the cost of testing, while maintaining the plasticity of the microbiota. Altogether, the findings of this study suggest that the combined administration can not only increase the pharmacological effect, as was seen by the reduction the MIC of the respective drugs, but can also minimize the side effects due to the administration of smaller doses, reducing the capacity to develop resistance to the therapy. Thus, the possibility of applying these molecules as synergistic potentiators of antibiotics and as a complementary therapeutic approach represents a promising future in the clinical–scientific scope for the treatment of highly resistant infectious diseases. However, as there are no reports on the use of a combination of lectins and antibiotics used in the clinical setting, further studies should be carried out to identify possible limitations, such as clinical trials to assess stability, dose, frequency, and selectivity, along with others. It is important to know whether the lectin can affect the drug’s molecular mechanism of action. Genetically modified organisms (GMOs) lacking the specific resistant mechanism can be used to define the pharmacokinetic and pharmacodynamic targets. Other aspects must be considered as well, such as polymicrobial infections, the presence of different resistance mechanisms, and combination with more than one antibiotic of different classes.

## Figures and Tables

**Table 1:** Lectins with antibacterial activity.

Target Microorganism	Lectin	Reference
*Escherichia coli*, *Klebsiella pneumoniae*, *Pseudomonas aeruginosa*, *Providencia stuartii*, ESBL, *Staphylococcus aureus*, MRSA, *Streptococcus mutans*, and *Enterococcus faecalis*	*Dypsis decaryi* (Ddel)	[39]
*Enterococcus faecalis*, *Pseudomonas aeruginosa* and *Staphylococcus aureus*	*Portulaca elatior* (PeRoL)	[40]
*Bacillus cereus*, *Bacillus subtilis*, *Enterococcus faecalis*, *Escherichia coli*, *Klebsiella pneumoniae*, *Micrococcus luteus*, *Pseudomonas aeruginosa*, *Salmonella enteritidis*, *Staphylococcus aureus*, *Streptococcus pyogenes*, and *Xanthomonas campestres*	*Apuleia leiocarpa* (ApulSL)	[41]
*Xanthomonas axonopodis* and *Clavibacter michiganensis*	*Acacia farnesiana* (AfaL)	[42]
*B. subtilis*, *K. pneumoniae*, *Staphylococcus epidermidis*, and *E. faecalis*	*Phthirusa pyrifolia* (PpyLL)	[43]

ESBL: extended-spectrum beta-lactamases; MRSA: oxacilin/methicillin-resistant *Staphylococcus aureus*.

**Table 2:** Antimicrobial activity of lectins alone and in combination with drugs.

Lectin	Microrganism	MIC	MBC	Synergism	Reference
*Canavalia ensiformis* (ConA)	MDR *S. aureus*	≥1024 μg/mL	NR	Gentamicin MICs were reduced in association with ConA (64 to 12.7 μg/mL)	[44]
*Canavalia ensiformis* (ConA)	MDR *E. coli*	≥1024 μg/mL	NR	Gentamicin MICs were reduced in association with ConA (32 to 20 μg/mL)	[44]
*Myracrodruon urundeuva* (MuBL)	*Staphylococcus aureus*	12.5 µg/mL	100.0 µg/mL	MuBL (0.4 μg/mL) and cefotaxime (0.2 μg/mL)	[45]
*Myracrodruon urundeuva* (MuHL)	*Staphylococcus aureus*	25.0 µg/mL	100.0 µg/mL	MuHL (0.8 μg/mL) and cefoxitin (0.2 μg/mL) MuHL (0.2 μg/mL) and cefotaxime (0.2 μg/mL)	[45]
*Myracrodruon urundeuva* (MuLL)	*Staphylococcus aureus*	25.0 µg/mL	100.0 µg/mL	MuLL (0.8 μg/mL) and cefoxitin (0.2 μg/mL) MuLL (0.4 μg/mL) and cefotaxime (1 μg/mL)	[45]
*Myracrodruon urundeuva* (MuBL)	CA-MRSA	25.0 µg/mL	100.0 µg/mL	MuBL (6.2 μg/mL) and cefoxitin (0.004 μg/mL) MuBL (6.2 μg/mL) and cefotaxime (0.004 μg/mL)	[45]
*Myracrodruon urundeuva* (MuHL)	CA-MRSA	25.0 µg/mL	100.0 µg/mL	MuHL (0.0007 μg/mL) and cefoxitin (32 μg/mL) MuHL (0.0007 μg/mL) and cefotaxime (32 μg/mL)	[45]
*Myracrodruon urundeuva* (MuLL)	CA-MRSA	50 µg/mL	100.0 µg/mL	MuLL (3.1 μg/mL) and cefoxitin (0.004 μg/mL) MuLL (6.2 μg/mL) and cefotaxime (0.004 μg/mL)	[45]
*Parkia platycephala* (PPL)	Multi-resistant *S. aureus*	≥1024 μg/mL	NR	Gentamicin MICs were reduced in association with DVL (64 to 25.4 μg/mL)	[32]
*Punica granatum* sarcotesta (PgTeL)	ESBL *Escherichia coli*	25 to 50.0 μg/mL	50 to 100.0 μg/mL	PgTel (0.003 to 0.48 μg/mL) and ceftazidime (0.78 to 12.5 μg/mL)	[46]
*Punica granatum* sarcotesta (PgTeL)	MBL *Escherichia coli*	25 μg/mL	100.0 μg/mL	PgTel (0.097 μg/mL) and ceftazidime (0.39 μg/mL)	[46]
*Punica granatum* sarcotesta (PgTeL)	ESBL *Escherichia coli*	25 to 50.0 μg/mL	50 to 100.0 μg/mL	PgTeL (6.25) and ampicillin (0.006 μg/mL)	[46]
*Punica granatum* sarcotesta (PgTeL)	ESBL *Escherichia coli*	25 to 50.0 μg/mL	50 to 100.0 μg/mL	PgTeL (0.0030 μg/mL) and carbenicillin (12.5 μg/mL)	[46]
*Punica granatum* sarcotesta (PgTeL)	MBL *Escherichia coli*	25 μg/mL	100.0 μg/mL	PgTeL (0.78 μg/mL) and cefuroxime (0.048 μg/mL)	[46]
*Calliandra surinamensis pinnulae* (CasuL)	*Staphylococcus* sp.	15.0 µg/mL	No activity	Casul (0.00183 μg/mL) and ampicillin (0.0156 μg/mL)	[47]
*Calliandra surinamensis pinnulae* (CasuL)	*Staphylococcus aureus*	3.75 µg/mL	No activity	Casul (2.30 × 10^−4^, 3.66 × 10^−3^ μg/mL) and tetracycline (0.12, 3.12 × 10^−2^ μg/mL)	[47]
*Alpinia purpurata* (ApuL)	MRSA	>400 µg/mL	No activity	ApuL (0.125 μg/mL to 50 μg/mL) and oxacillin (0.003 μg/mL to 7.7 μg/mL × 10^−6^)	[48]
*Alpinia purpurata* (ApuL)	MDR *Pseudomonas aeruginosa*	>400	No activity	ApuL (0.003 μg/mL) and ceftazidime (2 μg/mL)	[48]
Lectin from *Acinetobacter baumannii*	*Staphylococcus aureus*	256 µg/mL	No activity	Lectin and ceftazidime (32 µg/mL)	[49]
Lectin from *Acinetobacter baumannii*	*Escherichia coli*	1024 µg/mL	No activity	Lectin and ceftazidime (128 µg/mL)	[49]

MIC: minimum inhibitory concentration; MBC: minimum bactericidal concentration; CA-MRSA: community-acquired methicillin-resistant *Staphylococcus aureus*; MBL: metallo-β-lactamase; ESBL: extended-spectrum beta-lactamases; MDR: multidrug-resistant; NR: not reported.

**Table 3:** Lectins with antibiofilm activity.

Target Microorganism	Lectin	Reference
β-lactamase-producing *Escherichia coli*	*Punica granatum* (PgTeL)	[46]
*S. aureus* and *Candida albicans*	*Alpinia purpurata* (ApuL)	[48]
*Serratia marcescens* and *Bacillus* sp.	*Moringa oleífera* (WSMoL)	[66]
*S. aureus*, MRSA, *E. coli*, and *Staphylococcus saprophyticus*	*Calliandra surinamensis* (Casul)	[67]
*P. aeruginosa*	*Litchi chinensis*	[68]

ESBL: extended-spectrum beta-lactamases; MRSA: oxacilin/methicillin-resistant *Staphylococcus aureus*.

**Table 4:** Antibiofilm activity of lectins in combination with drugs.

Lectin	Microorganism	Antibiofilm Activity		Reference
		Alone	Combination	
Recombinant hemolymph plasma lectin (rHPL_OE_)	*Pseudomonas aeruginosa* PA14	rHPLOE at 0.63 μM inhibits 51% of *P. aeruginosa* biofilm formation rHPLOE at 5 μM inhibits 24% of preformed biofilm of *P. aeruginosa*	25 μM of azithromycin + rHPLOE at concentrations of 0.31, 0.63, 1.25 and 2.5 µM inhibit 19%, 21%, 39% and 43% of preformed biofilm of *P. aeruginosa*, respectively 25 µg/mL of cephalexin + rHPLOE at concentrations of 0.31, 0.63, 1.25 and 2.5 µM inhibit 33%, 33%, 38% and 50% of preformed biofilm of *P. aeruginosa*, respectively	[69]
*Calliandra surinamensis* pinnulae lectin (CasuL)	*Staphylococcus aureus*	CasuL at 3.75 µg/mL inhibits 30% of *S. aureus* biofilm formation	CasuL-tetracycline (0.00023 µg/mL + 0.12 µg/mL) inhibits approximately 26% of *S. aureus* biofilm formation CasuL-tetracycline (0.00366 µg/mL + 0.0312 µg/mL) inhibits approximately 60% of Ssp6PD biofilm formation CasuL-ampicillin (0.00183 µg/mL + 0.0156 µg/mL) inhibits approximately 35% of Ssp01 biofilm formation	[47]

## Data Availability

No new data were created or analyzed in this study. Data sharing is not applicable to this article.
